# Development of the Home-Based Fall Prevention Knowledge (HFPK) questionnaire to assess home-based fall prevention knowledge levels among older adults in China

**DOI:** 10.1186/s12889-022-14546-2

**Published:** 2022-11-12

**Authors:** Yuting Yang, Qiong Ye, Miao Yao, Yongwei Yang, Ting Lin

**Affiliations:** 1 The School of Nursing, Fujian Medical University, Fuzhou, Fujian China; 2 Nursing Department, the First Affiliated Hospital of Fujian Medical University, Fuzhou, Fujian China; 3 Ningde Municipal Hospital of Ningde Normal University, Ningde, Fujian China

**Keywords:** Older adults, Knowledge, Questionnaire development, Psychometric testing, Fall prevention

## Abstract

**Background:**

Falls are one of the main reasons for mortality and morbidity in older adults. However, despite adoption of prevention strategies, the number of falls in older adults has not declined. This study aimed to develop a questionnaire to assess fall prevention knowledge and explore associated factors among Chinese community-dwelling older adults.

**Methods:**

The Home-Based Fall Prevention Knowledge (HFPK) questionnaire was developed by Delphi expert consultation. We tested the internal consistency, reliability, and content validity of the HFPK. A total of 374 community-dwelling older adults participated in this study. The HFPK was used to assess their fall prevention knowledge.

**Results:**

After being evaluated by 15 experts, the item content validity index ranged from 0.867 to 1, and the scale content validity index was 0.985, which met the criterion for content validity. Cronbach’s α coefficient was 0.933, which satisfied the reliability criterion. Stepwise linear regression analysis showed that fall prevention knowledge was significantly associated with having higher education, being female, having a higher monthly income, people who were public officials before retirement, and having fewer children (*p* < 0.05).

**Conclusion:**

Fall prevention knowledge should be improved among older males and those with lower education, lower monthly income, people who were not public officials before retirement, and more children.

## Background

Globally, there were 703 million persons aged 65 or over in 2019. Eastern and South-Eastern Asia were regions with the largest elderly population, about 261 million people [1]. China, located in East Asia, has the largest elderly population. The seventh census [2] showed that at the beginning of November 2020, the number of people aged 60 and over in China was 264 million, accounting for 18.70% of the total population. Fall incidence in older adults continues to rise with ageing [3, 4]. About one-third of people older than 65 fall each year, and the incidence of falls in people above 80 is as high as 50% [5]. Among older adults, 71.20% will have contusions, abrasions, sprains, fractures, and even death after falling [6]. The annual incidence of injuries caused by falls is 11.58%. Falls have become the leading cause of injury among older adults and the second leading cause of death due to injury in older adults [7]. Jiayuan et al. [8] conducted a survey on the status of falls among 561 older adults in urban communities and found that the most commonplace falls were at home, accounting for 45.6%. Tolulope et al. [9] reviewed and analysed the case data of senile patients in the U.S. National Trauma Database from 2003 to 2006 and found that more than 42% of older adults fell at home. Therefore, preventing falls at home in older adults can help reduce the total rate of falls.

Fall prevention health education is a relatively inexpensive strategy, involving little activity, that helps reduce fall incidence [10, 11]. Most studies and reviews about fall prevention health education pay attention to older patients in hospital settings [12, 13] or nursing homes [14]. Many older individuals who live in urban neighbourhoods are still unaware of their odds of falling or deny their fall risk [15, 16]. However, initiation of awareness must begin with education [17]. It will raise the awareness and enrich the knowledge of older adults in recognising their fall threats so that fall incidence is reduced and early prevention achieved [18].

Evaluating older adults’ knowledge gaps is the initial step in implementing individualised and appropriate instruction [11]. However, previous studies mainly assessed fall prevention knowledge among older adults in nursing institutions [19] or in senile patients [20]. Despite some fall risk assessment scales or studies of fall prevention knowledge, beliefs, and behaviours, there is almost no high-quality tool specifically for assessing home-based fall prevention knowledge for older adults in the community domestically and overseas. For example, Morse [21] designed the Morse Fall Scale, which includes fall history for predicting the likelihood of falls in hospitalised patients in geriatric wards. Ying et al. [22] designed the Fall Prevention Knowledge, Belief, and Action Questionnaire that mainly explored these three aspects of fall prevention in hospitalised stroke patients. Although total reliability was 0.845 and content validity of the knowledge dimension was 0.98, specifics of the development process were lacking. In addition, there was no structural validity result as a reference, so its dimensions and item rationality still need to be verified. Yingfen et al. [23] designed a 16-item questionnaire on fall prevention knowledge and behaviour in senile hospitalised patients. Although fall prevention knowledge involved physiological function, disease, drug use, environment, fall knowledge source, and fall consequences, the questionnaire’s reliability and validity were also not reported. Although Hui et al. [24] developed an eight-item HFPK questionnaire for older adults living in communities, including environment, clothing, chronic diseases, seating, and drugs, the authors did not report the development process, reliability, and validity. Researchers were unaware of the efficacy of this questionnaire. In short, existing fall prevention knowledge questionnaires are mainly designed for hospitalised older adults. Some have not reported the development process, reliability, and validity, so the quality of the questionnaires is unknown. Therefore, the scientific nature of the questionnaire development process, rationality of the items, and their applicability in the older population need further exploring.

The content of tools for assessing falls at home and abroad is still not uniform. In 2014, the Morse Fall Assessment Scale, including five dimensions of physiology, psychology, pathology, biomechanics, and hospital environment, developed by Professor Janice Morse of Pennsylvania University was revised and introduced in China by Wenlan et al. [25]. It was applicable for hospitalized patients but did not consider the relationship between drugs and falls. Although Yu [26] designed the Knowledge, Belief, and Action Questionnaire for Falls Prevention to ask whether the long-term use of certain medications (such as antihypertensive drugs and sedatives) will increase the risk of falls, the questionnaire only lists two drugs. Other drugs that predispose to falls and are often taken by older adults, such as analgesics and psychotropic drugs, were not considered. In addition, although the questionnaire by Huimin [27] covered a wide range of fall prevention, including diseases, drugs, psychology, clothing, environment, and daily activities, it did not specifically list the types of drugs, which still need to be further improved. The Hendrich II Fall Risk Model was revised by Hendrich in Germany in 2003 [28] and revised by Congcong [29] in 2010 along three dimensions of conscious state, exertion pattern, and gait instability to assess hospitalized patients. However, this scale did not fully incorporate physical, psychological, disease, drug, and environmental factors that have proven influential factors in falls. The Home Falls and Accidents Screening Tool (HOME FAST) developed by Mackenzie et al. [30] in Australia was divided into two dimensions, including home environment and physical function among older adults, and was usually used to evaluate home-based fall risk factors in urban older adults. In 2015, Qiyun et al. [31] introduced the Chinese version of the HOME FAST and applied it to older adults in China. However, this questionnaire still contained two dimensions, including home environment and physical function among older adults, and no added other risk factors to analyse. The Fall Risk Assessment Scale for older adults in the community designed by Xiaona and Xiyun [32] was a 10-item scale without distinguishing dimensions. Its items included age, history of falls, level of cooperation, activity status, physical balance, gait, consciousness, illnesses, symptoms, and drug use. The above studies show that fall risk factors such as physiology, psychology, behaviour, and environment have attracted more attention, but there are few studies on the cognition, mental state, drugs, and lifestyle associated with falls.

Therefore, this study aimed to develop an HFPK questionnaire, including physiology, disease, lifestyle, environment, drug use, and mental, cognitive, and spiritual well-being to assess knowledge levels about fall prevention among urban older adults. This study started with literature research to determine the initial items of the HFPK. Under the guidance of experts through the Delphi method, the HFPK was revised scientifically. Then through factor analysis, we confirmed that the factor structure and the items met the criteria for a formal questionnaire with specific dimensions so that the HFPK has reasonable reliability and validity standards.

The Health Belief Model (HBM) was put forward in the 1950s [33], which pointed out that the formation of health beliefs played a pivotal role in people accepting these beliefs, improving their unhealthy behaviours, and adopting healthy behaviours. The HBM is based on needs and motivation theory, cognitive theory, and value expectation theory and uses social-psychological methods to explain the theoretical model of health-related behaviour [34]. It pays attention to people's attitudes and beliefs about health and internal and external factors that affect beliefs and emphasises subjective psychological processes of individuals, which include the effect of expectations, thinking, and beliefs on behaviours [35]. Therefore, the HBM was used to guide the development of the HFPK questionnaire.

## Methods and results

The study was conducted in three phases: (1) developing the HFPK, including constructing items, Delphi method, and extracting items; (2) testing the psychometric properties of the HFPK, including reliability and validity; and (3) understanding home-based fall prevention knowledge among urban older adults.

### Phase 1: developing the HFPK

#### Constructing items

We developed the raw HFPK to assess fall prevention knowledge based on the theoretical framework of the Health Belief Model and referred to the content of Expert Consensus on Fall Risk Assessment for the Elderly in China [36] and the Knowledge, Belief, and Action Questionnaire for Falls Prevention [26, 27].

#### Delphi method experts

Inclusion criteria for experts were as follows: (a) engaging in aged care, community care research, or related work; (b) working for more than 10 years; (c) having an intermediate-grade title or above; and (d) willing to participate in this research. From July to November 2020, we invited 15 nursing specialists from seven provinces across the country working in hospitals or schools to review our questionnaires. Among the experts, five were 30–39 years old, six were 40–49 years old, and four were over 50 years old. Experts with senior professional post titles accounted for 46.67%.

#### Delphi method round 1

Round 1 of the Delphi consisted of sending a copy of the 28-item questionnaire to the expert panel (*n* = 19) via electronic mail. Experts voted on a five-point scale (1 to 5, where 1 = very unimportant, and 5 = very important), which showed the extent to which they thought each of the 28 items needed to be included in the HFPK. Experts needed to return their responses within 2 weeks, and a reminder electronic mail was sent if no one responded after 1 week. Finally, 15 experts responded (response rate of 78.95%).

#### Delphi method round 2

Suggestions were collated in round 1. Then, the questionnaire was amended appropriately and sent to the same experts (*n* = 15) via e-mail in round 2 of the Delphi method. Experts needed to re-review the questions on the same five-point scale in light of results according to round 1. Experts still needed to return their responses within 2 weeks, and a reminder electronic mail was also sent if there was no response after 1 week. Finally, 15 experts responded (response rate of 100%).

#### Evaluation index

a. The response rate to the questionnaire reflects the extent of experts’ concern about the study. Generally, a response rate of 50% is the lowest proportion that can be analysed, 60% is a good result, and 70% is a better result [37]. In this study, the positive coefficient of Delphi round 1 was 78.95% and for Delphi round 2 was 100%.

b. Authority degree of expert opinion is expressed by authority coefficient (Cr), which is mainly based on self-evaluation, reflecting the reliability of the survey results [38]. Cr is determined by two factors, the expert judgement coefficient (Cα) and the expert familiarity coefficient (Cs). The relationship between them is Cr = (Cα + Cs)/3. The greater the Cr, the greater authority [39]. In this study, the Cr of Delphi round 1 was 0.86 and Delphi round 2 was 0.88.

c. The expert opinion coordination degree included the coefficient of variation (CV) and Kendall coordination coefficient (W). W reflects the consistency of different expert opinions. Generally, after two to three rounds of expert correspondence, W is significant after testing (*p* < 0.05), indicating that the expert evaluation opinions are good [38]. The smaller the CV, the higher the expert opinion coordination degree [38]. In general, the CV is ≤ 0.3, showing the variability of opinions among panellists is small [40]. In this study, the CV of Delphi round 1 was 0.12 and Delphi round 2 was 0.11. The W of Delphi round 1 was 0.191 and Delphi round 2 was 0.215.

#### Extracting items

After each round of the Delphi method, experts judged the significance of each item by Likert five-point scoring between 1 (completely unimportant) and 5 (completely important). Then, the items were extracted according to central tendency and discrete tendency. The central tendency was calculated by the number of experts who rated items with a score of 3 and above divided by the total number of experts. If rate was < 80%, it was be deleted [41]. The discrete tendency was calculated by the CV that the ratio of standard deviation, and the arithmetic average of item importance score, which if the CV is ≥ 0.2, it would be deleted [41].

In the Delphi method round 1, experts suggested adding 51, deleting 9, and revising 10 items. In round 2, they suggested adding 2, deleting 4, and revising 8 items. Therefore, the HFPK with 68 items was developed as shown in Table 1.

**Table 1:** Item-total correlation, factor loadings (*n* = 374) and independent samples *t*-test

Dimension	English items	Chinese items	*R*	Factor	*t*-value
Physiology & disease	1. Aging increases the risk of falls	1.年龄的增长会增加跌倒的风险	0.367*	0.556	-4.841*
	5. Decreased balance increases the risk of falls	5.平衡能力下降会增加跌倒的风险	0.392*	0.685	-5.618*
	6. Reduced muscle strength in the lower extremities increases the risk of falls	6.下肢肌力减退会增加跌倒的风险	0.421*	0.667	-5.027*
	7. Syncope increases the risk of falls	7.晕厥会增加跌倒的风险	0.315*	0.648	-4.266*
	10. Epilepsy can increase the risk of falls	10.癫痫会增加跌倒的风险	0.430*	0.507	-7.218*
	11. Parkinson's disease can increase the risk of falls	11.帕金森病会增加跌倒的风险	0.550*	0.500	-9.966*
	12. Alzheimer's increases the risk of falls	12.老年痴呆会增加跌倒的风险	0.499*	0.455	-10.097*
	13. Stroke increases the risk of falls	13.脑卒中会增加跌倒的风险	0.447*	0.510	-6.495*
	14. Hypotension increases the risk of falls	14.低血压会增加跌倒的风险	0.490*	0.484	-8.782*
	15. Hypertension can increase the risk of falls	15.高血压会增加跌倒的风险	0.533*	0.618	-9.872*
	16. Arrhythmias increase the risk of falls	16.心律失常会增加跌倒的风险	0.522*	0.459	-11.493*
Drug use	20. The use of psychotropic drugs increases the risk of falls	20.使用精神类药会增加跌倒的风险	0.580*	0.566	-15.461*
	21. The use of sedative pills increases the risk of falls	21.使用镇静安眠类药会增加跌倒的风险	0.604*	0.520	-16.757*
	22. The use of cardiovascular drugs increases the risk of falls	22.使用心血管药会增加跌倒的风险	0.494*	0.850	-9.770*
	23. The use of antiarrhythmic drugs increases the risk of falls	23.使用抗心律失常药会增加跌倒的风险	0.582*	0.850	-11.960*
	24. The use of antiepileptic drugs increases the risk of falls	24.使用抗癫痫药会增加跌倒的风险	0.537*	0.734	-11.859*
	25. Anti-dizziness drugs increase the risk of falls	25.抗晕动病药会增加跌倒的风险	0.454*	0.674	-9.190*
	26. The use of hypoglycemic drugs increases the risk of falls	26.使用降糖药会增加跌倒的风险	0.417*	0.597	-7.615*
	27. The use of central analgesics increases the risk of falls	27.使用中枢性镇痛药会增加跌倒的风险	0.524*	0.672	-9.633*
Mental, cognitive & spiritual well-being	28. Depression can increase the risk of falls	28.抑郁症会增加跌倒的风险	0.592*	0.580	-15.497*
	29. Anxiety can increase the risk of falls	29.焦虑症会增加跌倒的风险	0.637*	0.776	-20.653*
	30. Mania can increase the risk of falls	30.狂躁症会增加跌倒的风险	0.641*	0.907	-22.871*
	31. Phobias can increase the risk of falls	31.恐惧症会增加跌倒的风险	0.571*	0.909	-15.111*
	32. Obsession can increase the risk of falls	32.强迫症会增加跌倒的风险	0.588*	0.776	-17.210*
	33. Schizophrenia increases the risk of falls	33.精神分裂症会增加跌倒的风险	0.541*	0.692	-13.698*
	34. Low or impaired cognition can increase the risk of falls	34.认知低下或障碍会增加跌倒的风险	0.542*	0.540	-13.663*
Lifestyle & home environment	46. Getting up frequently at night increases the risk of falls	46.夜间频繁起夜会增加跌倒的风险	0.539*	0.483	-11.898*
	52. Cluttered indoor objects can increase the risk of falls	52.室内物品摆放杂乱会增加跌倒的风险	0.463*	0.607	-7.977*
	53. Lacking handrails in indoor activity venues can increase the risk of falls	53.室内活动场所缺乏扶手会增加跌倒的风险	0.528*	0.675	-10.203*
	54. High indoor stairs can increase the risk of falls	54.室内楼梯阶层过高会增加跌倒的风险	0.556*	0.484	-9.320*
	55. The lack of fall prevention signs on indoor stair steps can increase the risk of falls	55.室内楼梯台阶缺乏防跌标识会增加跌倒的风险	0.529*	0.635	-11.457*
	56. The turning corner of indoor stairs increases the risk of falls	56.室内楼梯转角过大会增加跌倒的风险	0.515*	0.458	-11.091*
	57. Lack of right-angle protectors at the edges or corners of indoor furniture can increase the risk of falls	57.室内家具边缘或转角处缺乏直角保护器会增加跌倒的风险	0.573*	0.527	-15.747*
	58. Uneven indoor floors can increase the risk of falls	58.室内地面不平整会增加跌倒的风险	0.401*	0.548	-4.641*
	59. High interior door thresholds increase the risk of falls	59.室内门槛过高会增加跌倒的风险	0.509*	0.612	-7.884*
	60. The height of the bed, seat, and sofa is too low or too high will increase the risk of falling	60.床铺、座椅、沙发高度过低或过高会增加跌倒的风险	0.540*	0.646	-13.128*
	61. Kitchen without non-slip tiles or non-slip mats will increase the risk of falls	61.厨房未铺防滑砖或防滑垫会增加跌倒的风险	0.546*	0.552	-10.010*
	62. Too much debris on the balcony can increase the risk of falls	62.阳台杂物过多会增加跌倒的风险	0.630*	0.621	-15.572*
	63. The lack of non-slip mats on the bathroom floor increases the risk of falls	63.卫生间地面缺乏防滑垫会增加跌倒的风险	0.473*	0.606	-6.349*
	64. Lack of handrails next to the bathroom or toilet increases the risk of falls	64.浴室或马桶旁缺乏扶手会增加跌倒的风险	0.542*	0.575	-12.601*
	66. Lack of seats or weak seats in the shower increases the risk of falls	66.淋浴间缺乏座椅或座椅不牢固会增加跌倒的风险	0.491*	0.625	-10.728*
	67. Not wearing slippers during shower increases the risk of falling	67.淋浴时未穿防滑拖鞋会增加跌倒的风险	0.350*	0.641	-4.991*
	68. Dry and wet toilet without separation can increase the risk of falls	68.卫生间干湿未分离会增加跌倒的风险	0.500*	0.668	-10.046*
Deleted items	2. Vision loss increases the risk of falls	2.视力减退会增加跌倒的风险		-	
	3. Hearing loss can increase the risk of falls	3.听觉减退会增加跌倒的风险		-	
	4. Decreased sense of touch can increase the risk of falls	4.触觉减退会增加跌倒的风险		-	
	8. Pain episodes can increase the risk of falls	8.疼痛发作会增加跌倒的风险		-	
	9. Poor sleep quality can increase the risk of falls	9.睡眠质量不好会增加跌倒的风险		-	
	17. Osteoarthritis can increase the risk of falls	17.骨关节炎会增加跌倒的风险		-	
	18. Osteoporosis can increase the risk of falls	18.骨质疏松会增加跌倒风险		-	
	19. Cervical spondylosis increases the risk of falls	19.颈椎病会增加跌倒风险		-	
	35. Overestimation of your condition increases the risk of falls	35.对自身状况的高估会增加跌倒的风险		-	
	36. Insufficient awareness of fall risk increases the risk of falls	36.对跌倒风险认识不足会增加跌倒的风险		-	
	37. Decreased responsiveness increases the risk of falls	37.反应敏捷度下降会增加跌倒的风险		-	
	38. Fear of falling increases the risk of falling	38.害怕跌倒会增加跌倒的风险		-	
	39. A history of falls within 6 months increases the risk of falls	39.6个月内有跌倒史会增加跌倒的风险		-	
	40. Long clothes and pants can increase the risk of falls	40.衣服裤子过长会增加跌倒的风险		-	
	41. Improperly sized or non-slip shoes can increase the risk of falls	41.鞋子大小不合适或不防滑会增加跌倒的风险		-	
	42. Wearing too much clothing can increase the risk of falls	42.衣物穿着过多会增加跌倒的风险		-	
	43. A sudden change of position increases the risk of falls	43.突然改变体位会增加跌倒的风险		-	
	44. Climbing for objects can increase the risk of falling	44.登高取物会增加跌倒的风险		-	
	45. Lifting heavy objects increases the risk of falling	45.提重物会增加跌倒的风险		-	
	47. Exercise increases the risk of falls	47.运动锻炼会增加跌倒的风险		-	
	48. Poor indoor lighting can increase the risk of falls	48.室内光线不良会增加跌倒的风险		-	
	49. Poor indoor ventilation can increase the risk of falls	49.室内通风不良会增加跌倒的风险		-	
	50. A slippery indoor floor increases the risk of falls	50.室内地面湿滑会增加跌倒的风险		-	
	51. Small indoor spaces can increase the risk of falls	51.室内空间狭小会增加跌倒的风险		-	
	65. Using a bathtub increases the risk of falls	65.使用浴缸会增加跌倒的风险		-	

Note. “*”: *p* < 0.001; “-”: Factor loadings were lower than 0.45; 68 items belong to the FHPK under revision, in which participants were filled out in the order of items marked

### Phase 2: testing the psychometric properties of the HFPK

#### Participants

The recommended sample size is 5 to 10 participants per item. The number of the items was intended to be 68, so the sample size estimated was 340 to 680 [42]. Considering a 10% non-response rate, the minimum sample size was set at 378 participants. A total of 374 people in a convenience sample were recruited from Hot Spring Apartments, Hualin Garden, Fenghu New Town, and Dongtang Community in the Gulou District in Fuzhou, China, between January and March 2021. The response rate was 98.94%. Inclusion criteria included age older than 60, living in an urban community for 6 months or more, ability to express and comprehend simple Chinese characters, and willingness to complete questionnaires. Exclusion criteria were hearing impairment, speech impairment, severe cognitive impairment, mental illness, and other severe or terminal diseases.

#### Measures

The revised HFPK comprises 68 items grouped into five subscales, including physiology and disease (19 items, 0–19 score), drug use (8 items, 0–8 score), mental, cognitive, and spiritual well-being (12 items, 0–12 score), lifestyle (8 items, 0–8 score), and home environment (21 items, 0–21 score). Scores range from 0 to 68. All items are not reverse-scored, with three responses to yes, uncertain, and no, which are all multiple-choice questions. If the answer is yes, the response is considered correct and scores 1. If the answer is unclear or no, the response is incorrect and scores 0. The higher the score, the higher the knowledge level of older adults in preventing falls at home. According to the 100-point scale, 85 ~ 100 is excellent, 75 ~ 84 is good, 60 ~ 74 is medium, and below 60 is poor [43]. The revised HFPK was categorized as follows: 58 ~ 68 as excellent, 51 ~ 57 as good, 41 ~ 50 as medium, and below 40 as poor [44].

#### Data collection

Before the collection stage, the Ethical Review Committee of Fujian Medical University approved this study. The investigators, who were five graduate students and several undergraduates from Fujian Medical University, were uniformly trained with an emphasis on the use of uniform instructions. The sites surveyed were in the homes of older adults in their communities or other quiet places that followed their wishes. The investigators recruited subjects face-to-face and asked older adults if they would like to participate in this study. Then, they informed participants of the research purpose, significance, and privacy rights and conducted one-to-one questionnaire surveys after participants signed written informed consent.

In the formal investigation stage, each questionnaire was conducted through face-to-face interviews between trained investigators and respondents. The investigators informed older adults of the purpose of filling out the different questionnaires and the meaning of the options in advance. Investigators asked every item in the questionnaire in a neutral, unbiased manner, as most of the respondents were old and had relatively low education, to ensure the consistency of participants' understanding of the questionnaire and to improve the response rate. Investigators filled out the questionnaire accordingly after ensuring that the respondents understood the question and gave their answers independently. Then, they immediately checked it after finishing the questionnaire to avoid incompleteness due to non-compulsive reasons such as forgetting to reply or temporarily needing to think. If participants were unwilling to answer some questions, investigators would not force them to answer but would continue to ask other follow-up questions. Generally, participants could fill in the questionnaire in 20–30 min. After completing the questionnaire, researchers presented participants with souvenirs and expressed gratitude.

After collecting the data, the researchers stored the participants' identity information separately from the questionnaire content. The identity information could be mapped one-to-one by number to identify the corresponding questionnaire. All researchers kept the identities of participants confidential. Researchers were not allowed to reveal their identifiable information to anyone without permission. Identity information files were kept in locked filing cabinets and viewed only by researchers. When necessary, members of the government management department or ethics committee could consult the data according to regulations at the research department.

#### Data analysis

Statistical Product Service Solutions (SPSS) version 26.0 (Armonk, New York) was used for assessing the HFPK regarding content validity and exploratory factor analysis (EFA). IBM SPSS Amos version 24.0 (IBM Corporation, Armonk, NY, USA) was used to conduct confirmatory factor analysis (CFA). This study was tested for validity in the following order: (1) Content validity: Content validity can be expressed by the content validity index (CVI), including the item content validity index (I-CVI) and scale content validity index (S-CVI). An I-CVI above 0.78 and S-CVI above 0.90 were considered to meet the criteria of CVI [45]. (2) Construct validity: The same 374 participants were used to analyse EFA and CFA, verifying construct validity. EFA was used to assess the underlying construct of the items by using principal axis factoring with Promax rotation. Before conducting factor analysis, it was necessary to examine the applicability of the analysis. A Kaiser–Meyer–Olkin index (range from 0 to 1) greater than 0.50 and the result of the Bartlett test of sphericity were considered as eligible for performing EFA [46]. The following criteria were used to determine the number of valuable factors: (a) eigenvalues greater than 2.0 [47], (b) Cattell scree plot, (c) the percentage of total explained variance accounted for, and (d) items with loadings greater than 0.45 in absolute value [48]. Then, CFA was conducted to confirm the factorial structure identified in the exploratory study. Parameters in the model were estimated by maximum likelihood estimation. Parameters [49, 50] used to test the goodness of fit of the model included the ratio of the *χ2* value and the degrees of freedom (*χ2*/*df*), Goodness of Fit Index (GFI), Adjusted Goodness of Fit Index (AGFI), Comparative Fit Index (CFI), Tucker Lewis Index (TLI), Normed Fit Index (NFI), Standardised Root Mean Square Residual (SRMR) and Root Mean Square Error Approximation (RMSEA). Model fit was deemed acceptable as the following values were met: *χ2*/*df* < 3.0 [51]; GFI > 0.80; AGFI > 0.80 [52]; TLI > 0.90; NFI > 0.90; SRMR < 0.08 [53]; RMSEA < 0.08; and CFI > 0.90 [50].

## Results

### Demographic characteristics of participants

There were 378 questionnaires distributed to older adults in urban communities, 374 of the recovered questionnaires were valid, and the response rate was 98.94%. Although investigators gave participants a detailed introduction to this research’s purpose and significance and conducted a face-to-face questionnaire collection at the beginning of the survey, four participants indicated they were unwilling to continue participating because of the long time to complete the questionnaire. We respected the right of participants to withdraw from this research at any time. The demographic characteristics of participants are shown in Table 2.

**Table 2:** Demographic characteristics of participants

Variable	Categories	*n*	%
Age, year	60 ~ 69	182	48.66
	70 ~ 79	128	34.22
	≥ 80	64	17.11
Gender	Male	188	50.27
	Female	186	49.73
Education	Illiteracy	35	9.36
	Primary school	64	17.11
	Junior high school	81	21.66
	High school or technical secondary school	95	25.40
	Junior college	38	10.16
	Undergraduate and above	61	16.31
Marital status	Married	348	93.04
	Single	1	0.27
	Widowed	24	6.42
	Divorced	1	0.27
Monthly personal income (RMB)	< 1000	53	14.17
	1000 ~ 2000	42	11.23
	2000 ~ 4000	131	35.03
	≥ 4000	148	39.57
Number of chronic diseases	None	154	41.18
	1	139	37.17
	2	60	16.04
	≥ 3	21	5.61
Self-care	cannot take care of themselves	3	0.80
	Partly self-care	18	4.81
	Completely self-care	353	94.39
Children number	0	3	0.80
	1	164	43.85
	2	134	35.83
	≥ 3	73	19.52
Whether the children live together	Live in the same city but not together	133	35.56
	do not live in the same city	35	9.36
	living together	206	55.08
Primary caregiver	Spouse	59	15.78
	Nanny	9	2.41
	Child	25	6.68
	Self-care	281	75.13
Occupation before retirement	Worker	109	29.14
	farmer	65	17.38
	teacher	22	5.88
	civil servant	56	14.97
	medical staff	10	2.67
	Self-employed and others	112	29.95
Smoking frequency	never	333	89.04
	1–2 days a week	6	1.60
	3–4 days a week	7	1.87
	Almost every day	28	7.49
Drinking frequency	never	302	80.75
	1–2 days a week	41	10.96
	3–4 days a week	11	2.94
	Almost every day	20	5.35
Exercise frequency	never	35	9.36
	1–2 days a week	43	11.50
	3–4 days a week	45	12.03
	Almost every day	251	67.11

### Content validity

After being evaluated by 15 experts, I-CVI ranged from 0.867 to 1, and S-CVI was 0.985, which achieved the criteria for content validity, showing that the questionnaire items were accurate and comprehensive [45].

### Construct validity

#### Exploratory factor analysis

**Fig.1:**
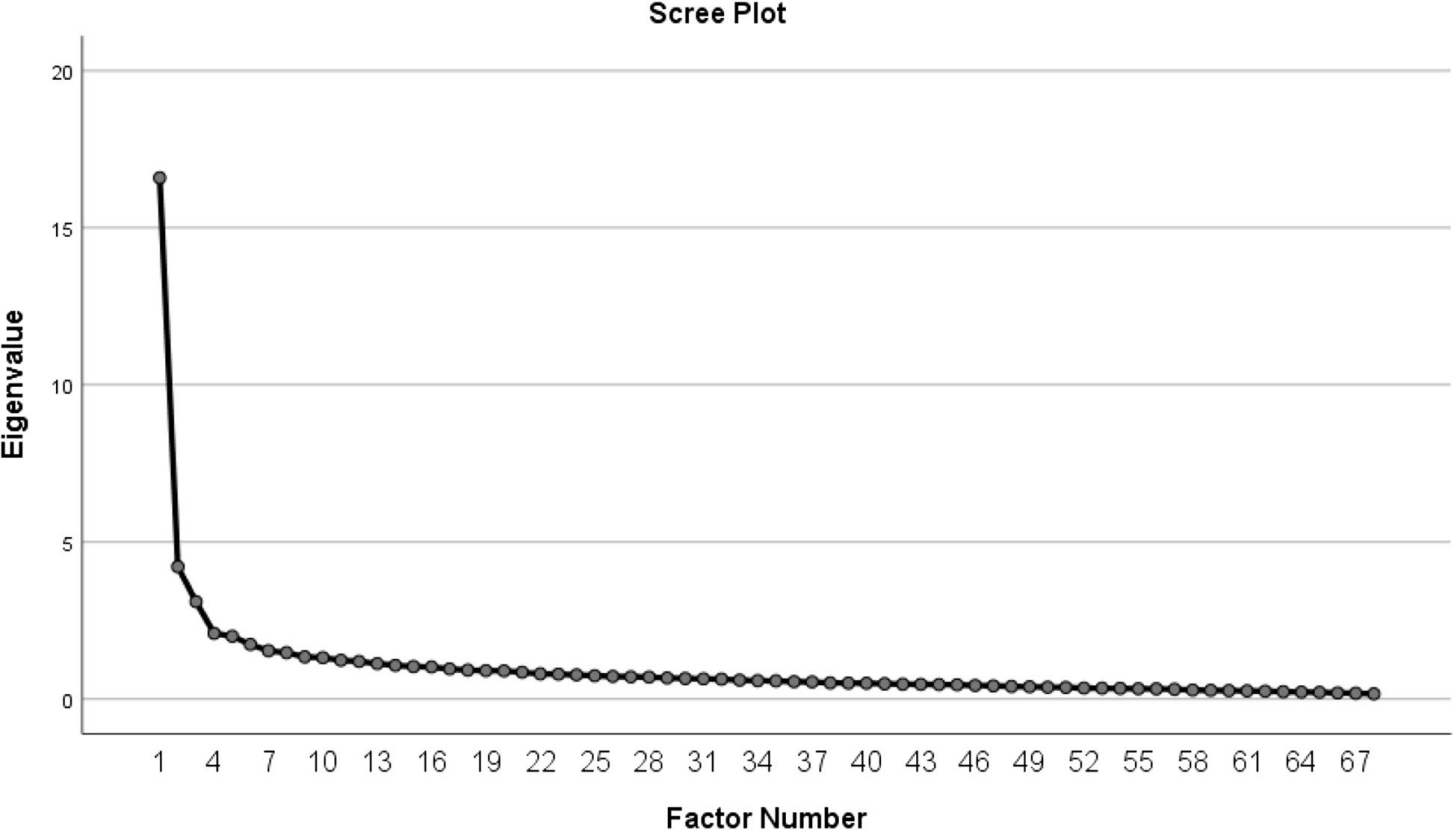
Cattell scree plot

The *Kaiser–Meyer–Olkin* value was 0.918, and the significance of Bartlett’s test of sphericity was less than 0.001 (*χ2* = 11,230.264, *df* = 2278). These results indicate using factor analysis was appropriate. Principal axis factoring with Promax rotation assessed the underlying construct of the items. Four factors were confirmed with eigenvalues greater than 2.0, and a total explained variance was 38.23% (Table 3). The factor loadings of all items were higher than 0.45 (Table 1). Examination of the scree slope identified that a four-component solution was suitable (Fig. 1).

**Table 3:** Explained variance of each factor (*n* = 374)

Component	Eigenvalue	Explained Variance (%)	Cumulative Explained Variance (%)
1	16.588	24.394	24.394
2	4.213	6.196	30.590
3	3.100	4.559	35.149
4	2.090	3.074	38.223

#### Confirmatory factor analysis

**Fig. 2:**
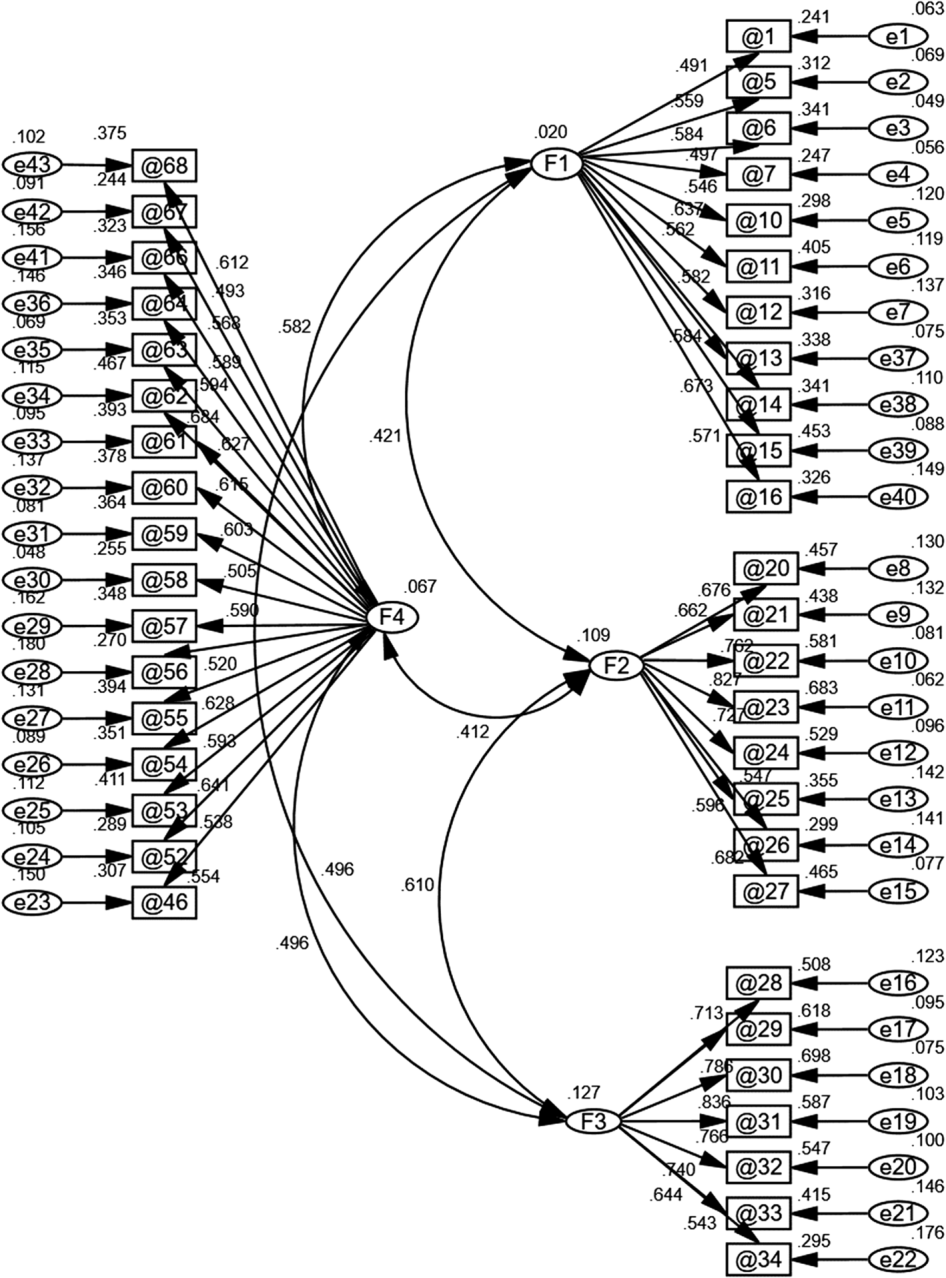
Confirmatory factor analysis standardized correlation diagram (*n* = 374)

CFA was performed using the IBM SPSS Amos version 24.0 to obtain a four-factor structural equation model as shown in Table 4. Inadequate individual indicators were not enough to reject the model [51]. Investigators considered that the model could be accepted. Figure 2 shows that the standardised factor loadings, ranging from 0.491 to 0.827, were statistically significant in the four-factor model.

**Table 4:** Model-fit index of confirmatory factor analysis (*n* = 374)

Inspected Fit Indices	Acceptable Fit	Confirmatory Factor Analysis Fit Indices	Degree of Fit
*χ*^*2*^/*df* (*χ*^*2*^ = 1820.868, *df* = 854)	< 3.0	2.132	Well
SRMR	< 0.08	0.062	Well
RMSEA	< 0.08	0.055	Well
GFI	> 0.80	0.805	Well
AGFI	> 0.80	0.784	General
CFI	> 0.90	0.850	General
TLI	> 0.90	0.841	General
NFI	> 0.90	0.752	General

### Phase 3: understanding HFPK among urban older adults

#### Measures

The revised HFPK was adjusted to a 43-item formal HFPK with four dimensions via construct validity. They are physiology and disease (11-item), drug use (8-item), mental, cognitive, and spiritual well-being (7-item), and lifestyle and home environment (17-item). Each item has a score of 1 or 0, so the highest total is 43, and the lowest is 0, with three responses of yes, no, and uncertain. Yes gets 1. No or unclear gets 0. The scores indicate how much older adults know about preventing falls at home. Similar to the revised HFPK described above, the meaning of the scores of the formal HFPK is as follows. 37 ~ 43 scores represent excellently, 33 ~ 36 scores mean good, 26 ~ 32 scores show medium, and below 25 scores are poor.

#### Data analysis

Based on Phase 1 and Phase 2, researchers obtained a formal HFPK with good content validity and construct validity for 48 items. Then, researchers verified the item discrimination and reliability of the formal HFPK. Statistical Product Service Solutions (SPSS) version 26.0 (Armonk, New York) was used for item analysis and reliability analysis. (1) Item analysis: When we develop a new measure, carrying out sufficient pilot work is significant because it can identify items that lack clarity or may not be appropriate for or without discrimination between participants. Item analysis is one way to pilot a new questionnaire, which provides a series of simple heuristic methods for retaining or deleting items [54]. Item analysis included the Pearson correlation analysis and independent samples *t*-test. The former assesses item-total correlations, and the latter evaluates the degree of discrimination of the items. The standard advice is to eliminate an item whose item-scale correlation was < 0.30 [55]. Scores were rank-ordered, and 27% of the highest and lowest scorers were selected for the independent samples *t*-test, comparing statistical differences between two groups [56] for item discrimination. (2) Reliability analysis: Researchers calculated the internal consistency reliability to determine stability of the questionnaire. The higher Cronbach’s *α* total coefficient, calculated to examine internal consistency, means higher reliability. Generally, Cronbach’s *α* total coefficient is considered acceptable if higher than 0.80 [55].

The home-based fall prevention knowledge levels and Influencing factors among urban older adults were reported in Phase 3 via a cross-sectional survey. Statistical Product Service Solutions (SPSS) version 26.0 (Armonk, New York) was used for descriptive statistics and multiple linear regression analysis. (1) Descriptive statistics were used to describe scores on four dimensions (physiology and disease; drug use; mental, cognitive, and spiritual well-being; lifestyle and home environment) and the total score of the home-based fall prevention knowledge levels among urban older adults. (2) Multiple linear regression analysis was used to explore the influence of the independent variable on the dependent variable. Independent variables were demographic characteristics. Dependent variables were four dimensions and the total score of the home-based fall prevention knowledge. With the stepwise regression method adding factors, independent variables entered in the model if the difference was statistically significant (*p*-values < / = 0.05 for the *F*-test) and were excluded if without statistically contributing to the prediction of the dependent variable (*p*-values > / = 0.10 for the *F*-test). Researchers used the standardized coefficient *Beta* to judge the effect size of each factor and applied the coefficient of determination (*R*^*2*^) and its adjusted version to test goodness of fit. *p* < 0.05 stated a statistically significant difference with 0.05 as the significance level.

## Results

### Item analysis

The item-total correlation was used for Pearson correlation analysis [55]. We found that each item score was positively related to the total one, and item-total correlations ranged between 0.315 and 0.641 (*p* < 0.001) (Table 1), showing that 48 items with homogeneity can be preserved. Testing if the difference was meaningful between the HFPK scores of the 27% higher (*n* = 108) and 27% lower (*n* = 115) ranges after the raw scores were ranked from small to large judged the item discrimination [56]. The difference between the two groups for each item was used for independent samples *t*-test. We found that the statistical value of each item was statistically significant (*p* < 0.001), and ideal item discrimination was present, as shown in Table 1.

### Reliability

Cronbach’s *α* was calculated to examine internal consistency of the formal HFPK. Cronbach’s *α* coefficients for the four factors and the total scale were 0.836 (physiology and disease), 0.875 (drug use), 0.881 (mental, cognitive, and spiritual well-being), 0.896 (lifestyle and home environment), and 0.933 (total scale), respectively, indicating adequate internal consistency [57].

### Descriptive statistics and multiple linear regression analysis results

Scores on dimensions and total scores on HFPK are shown in Table 5.

**Table 5:** Dimensions scores and total score of home-based fall prevention knowledge

Dimension	Score (‾x ± s)	Correct rate (%)
Physiology & disease (range of scores: 0 ~ 11)	8.99 ± 2.56	81.77
Drug use (range of scores: 0 ~ 8)	2.36 ± 2.64	29.51
Mental, cognitive & spiritual well-being (range of scores: 0 ~ 7)	3.20 ± 2.65	45.76
Lifestyle & home environment (range of scores: 0 ~ 17)	12.65 ± 4.41	74.39
Total score (range of scores: 0 ~ 43)	27.21 ± 9.49	63.27

In the linear regression, as shown in Table 6, 3.5% of this model would explain the variance of the dependent variable, total knowledge level (adjusted *R*^*2*^ = 0.035; *p* < 0.05). Education would be a predictive factor of this model (*β* = 0.195; *p* < 0.001).

**Table 6:** Regression model: socio-demographic variables and total score

**Model**	***R***	***R***^***2***^	**Adjusted *R***^***2***^	**Standard Error of the Estimate**	**ANOVA**		
					***F***		**Sig. *F***
1	0.195	0.038	0.035	9.320	14.656		< 0.001
**Model 1**	**Unstandardised Coefficients**		**Standardised Coefficients**		**95% Confidence Interval for *B***		
	***B***	**Std. Error**	***Beta***	***t***	***p***	**Lower Bound**	**Upper Bound**
(Constant)	22.867	1.232	-	18.567	< 0.001	20.445	25.289
Education	1.209	0.316	0.195	3.828	< 0.001	0.588	1.830

*Note. R* = coefficient of determination; *F* = Fisher-Snedecor test; *β* = Regression coefficient; *t* = Student’s t-test. Correlation is significant at the 0.05 level

As shown in Table 7, three models were generated, with model 3 having the largest predictive capacity for physiology and disease knowledge variability (adjusted *R*^*2*^ = 0.083; *p* < 0.05). The significant variables that explain the model negatively, i.e., each of these variables decreased physiology and disease knowledge levels irrespective of other variables, were as follows: number of children (*β* =  − 0.159; *p* = 0.002) and gender (*β* =  − 0.131; *p* = 0.010). On the other hand, education was associated with increased physiology and disease knowledge levels irrespective of other variables (*β* = 0.206; *p* < 0.001). According to the ANOVA test, the linear relationship was significant between the dependent variable and the set of independent variables of this model (*F* = 12.193; *p* < 0.001).

**Table 7:** Regression model: socio-demographic variables and physiology & disease dimension

**Model**	***R***	***R***^***2***^	**Adjusted *R***^***2***^	**Standard Error of the Estimate**	**ANOVA**		
					***F***		**Sig. *F***
1	0.226^a^	0.051	0.049	2.494	20.013		< 0.001
2	0.271^b^	0.073	0.068	2.468	14.709		< 0.001
3	0.300^c^	0.090	0.083	2.449	12.193		< 0.001
**Model 3**	**Unstandardised Coefficients**		**Standardised Coefficients**		**95% Confidence Interval for *B***		
	***B***	**Std. Error**	***Beta***	***t***	***p***	**Lower Bound**	**Upper Bound**
(Constant)	9.523	0.651		14.631	< 0.001	8.243	10.802
Education	0.344	0.089	0.206	3.883	< 0.001	0.170	0.518
Children's number	-0.520	0.170	-0.159	-3.054	0.002	-0.854	-0.185
Gender	-0.670	0.259	-0.131	-2.590	0.010	-1.179	-0.161

*Note. R* = coefficient of determination; *F* = Fisher-Snedecor test; *β* = Regression coefficient; *t* = Student’s t-test; a = education; b = education, children number; c = education, children number, gender. Correlation is significant at the 0.05 level

As shown in Table 8, 1.4% of this model explained the variance of the dependent variable, drug use knowledge level (adjusted *R*^*2*^ = 0.014; *p* < 0.05). Education was a predictive factor of this model (*β* = 0.130; *p* = 0.012).

**Table 8:** Regression model: socio-demographic variables and drug use dimension

**Model**	***R***	***R***^***2***^	**Adjusted *R***^***2***^	**Standard Error of the Estimate**	**ANOVA**		
					***F***		**Sig. *F***
1	0.130	0.017	0.014	2.621	6.394		0.012
**Model 1**	**Unstandardised Coefficients**		**Standardised Coefficients**			**95% Confidence Interval for *B***	
	***B***	**Std. Error**	***Beta***	***t***	***p***	**Lower Bound**	**Upper Bound**
(Constant)	1.555	0.346	-	4.488	< 0.001	0.874	2.236
Education	0.225	0.089	0.130	2.529	0.012	0.050	0.399

*Note. R* = coefficient of determination; *F* = Fisher-Snedecor test; *β* = Regression coefficient; *t* = Student’s t-test. Correlation is significant at the 0.05 level

As shown in Table 9, 2.8% of this model explained the variance of the dependent variable, mental, cognitive, and spiritual well-being knowledge level (adjusted *R*^*2*^ = 0.028; *p* < 0.05). Occupation before retirement was a predictive factor of this model (*β* = 0.174; *p* = 0.001).

**Table 9:** Regression model: socio-demographic variables and mental, cognitive & spiritual well-being dimension

**Model**	***R***	***R***^***2***^	**Adjusted *R***^***2***^	**Standard Error of the Estimate**	**ANOVA**		
					***F***		**Sig. *F***
1	0.174	0.030	0.028	2.612	11.649		0.001
**Model 1**	**Unstandardised Coefficients**		**Standardised Coefficients**			**95% Confidence Interval for *B***	
	***B***	**Std. Error**	***Beta***	***t***	***p***	**Lower Bound**	**Upper Bound**
(Constant)	2.948	0.154	-	19.087	< 0.001	2.644	3.251
Occupation before retirement	1.087	0.318	0.174	3.413	0.001	0.461	1.713

*Note. R* = coefficient of determination; *F* = Fisher-Snedecor test; *β* = Regression coefficient; *t* = Student’s t-test. Correlation is significant at the 0.05 level

As shown in Table 10, 2.2% of this model explained the variance of the dependent variable, lifestyle and home environment knowledge level (adjusted *R*^*2*^ = 0.022; *p* < 0.05). Monthly personal income was a predictive factor of this model (*β* = 0.157; *p* = 0.002).

**Table 10:** Regression model: socio-demographic variables and lifestyle & home environment dimension

**Model**	***R***	***R***^***2***^	**Adjusted *R***^***2***^	**Standard Error of the Estimate**	**ANOVA**		
					***F***		**Sig. *F***
1	0.157	0.024	0.022	4.357	9.341		0.002
**Model 1**	**Unstandardised Coefficients**		**Standardised Coefficients**			**95% Confidence Interval for *B***	
	***B***	**Std. Error**	***Beta***	***t***	***p***	**Lower Bound**	**Upper Bound**
(Constant)	10.655	0.690	-	15.447	< 0.001	9.298	12.011
Monthly personal income	0.664	0.217	0.157	3.056	0.002	0.237	1.091

*Note. R* = coefficient of determination; *F* = Fisher-Snedecor test; *β* = Regression coefficient; *t* = Student’s t-test. Correlation is significant at the 0.05 level

## Discussion

### Scientific and normative development process

First, based on the consensus of experts [36] and existing studies [26, 27], the risk factors related to falls at home [58] were extracted into the item pool for enriching HFPK content. Second, the HFPK underwent two rounds of Delphi expert consultation. All experts were employed in geriatric nursing or community nursing from different provinces across the country. All indicators of the results of Delphi expert consultation were good, indicating experts have high enthusiasm for this subject, and the results of the consultation are reliable. Third, we extracted four common factors and determined four dimensions and 43 items through EFA and CFA. The structure of the formal HFPK is therefore reasonable.

### Reliability and validity of the HFPK

In this study, the HFPK was sufficiently homogeneous with accurate item discrimination and had satisfactory reliability, construct validity, and content validity for use among urban older adults in China. First, under the guidance of experts, the revised raw HFPK was endorsed by 15 experts. Both I-CVI and S-CVI results suggest that the HFPK has good content validity. Second, principal axis factoring analysis showed that the scale has a clear structure and that the attribution of each item is consistent. Each item factor loading was above 0.45, and the cumulative contribution rate of the four factors was 38.223%. Because each dimension is clear and explanatory, the scale has good construct validity. The model fits the observed data well and supports EFA, which also validates construct validity. Third, item analysis examined the quality of the HFPK, showing that it had good item discrimination, preserving 43 items extracted out by factor analysis. Finally, it demonstrated that the scale has satisfactory internal consistency, according to results of a study by Polit and Beck [55]. The total Cronbach's α coefficient of the HFPK in older adults was 0.933, and for the four subscales was 0.836 (physiology and disease), 0.875 (drug use), and 0.881 (mental, cognitive, and spiritual well-being), 0.896 (lifestyle and home environment). Cronbach's α coefficients of the knowledge dimension in the fall prevention questionnaires designed by Ying et al. [22] and Liping et al. [59] were 0.845 and 0.772, indicating that the internal consistency of the two questionnaires was good, but both were lower than in this study. This may be related to the fact that the questionnaire designed in this study has more items and richer content. The internal construct of the HFPK was relatively stable when tested with older adults and external validity was good.

### Home-based fall prevention knowledge levels among urban older adults

This study showed that the average score of older adults' HFPK is 27.21 ± 9.49 scores and the correct rate of the total score is 63.27%, indicating that urban older adults have a moderate grasp of fall prevention knowledge. Older adults had uneven and insufficient knowledge about understanding all aspects of HFPK, in which physiology and disease, lifestyle, and home environment better than drug use and mental, cognitive, and spiritual well-being. Liping et al. [59] concluded that older adults' awareness rate of the home environment and requirements to prevent falls is 70%. In addition, Ying et al. [22] found that older adults have a higher rate of correct answers in judging fall- susceptible populations and the environment. This study is consistent with the above findings. Older adults grasped fall prevention knowledge about physiology, disease, lifestyle, and home environment well, which may be associated with increasing concerns about their health and the safety of their home environment. However, urban older adults did not understand the relationship of drug use and mental, cognitive, and spiritual well-being to falls enough. A prospective study [60] assessed 488 community-based older adults aged 70 years and above with comprehensive psychological, cognitive, and physical items and found that depressive symptoms and antidepressant use were significant contributing factors to falls. Negative emotions increased fall risk because older adults with loneliness or depression had many accompanying symptoms, including cognitive impairment, slow response, low muscle strength in the lower limbs, and even abnormal mental states [61–63]. Taking many other drugs that promote sleep and excretion of urine and lower blood pressure and blood sugar could also enhance the risk of falls in addition to antidepressants [64, 65]. However, the awareness rate of drug knowledge among older adults was lower than 30% [59], which is consistent with the findings of this study, indicating that most older adults are not aware that drugs may easily cause falls. Therefore, it is necessary to enhance the education of older adults about psychological and drug-related factors of falls.

### Influencing factors on HFPK among urban older adults

HFPK was related to education, gender, monthly income, occupation before retirement, and number of children. First, the greater the education, the more channels people had for obtaining knowledge. Education is one of the factors influencing fall prevention knowledge in older adults [65, 66], which is consistent with this study. Therefore, we could use less text and use more pictures, animations, or videos to improve knowledge on preventing falls in older adults with low education. Second, gender is one of the factors influencing knowledge of physiology and disease among older adults, and men scored lower than women did, which was consistent with findings of Mingyan et al. [67]. Compared with men, women have a higher incidence of osteoporosis and are more likely to fall, so they pay more attention to prevention and acquisition of fall prevention knowledge. Third, the higher the monthly income, the greater the knowledge of lifestyle and home environment factors, which was consistent with the study of Liujing et al., [65]. With better economic conditions, older adults pay more attention to their quality of life and preventing accidents such as falls. Fourth, people who were public officials before retirement had a richer knowledge of mental, cognitive, and spiritual well-being factors. They may be of higher social status, more respected, and more aware of the importance of mental health to avoid events such as falls. Finally, the fewer children, the higher fall prevention knowledge of physiology and disease factors. It may be that older adults who have no children or have few children are less dependent. They pay more attention to their physiological diseases, become more self-reliant in life behaviour, and have a higher incentive to acquire fall prevention knowledge.

### Limitations

This study has some limitations. First, we based it on the principle of reporting a kind of reliability and a kind of validity. The HFPK is anonymous, so it is impossible to repeat the measurement for the same participant. Therefore, this study cannot examine the retest reliability or other reliabilities of the HFPK. In addition, the study also did not delve into the results of other validities, such as criterion validity. Second, although we included as many fall-related risk factors as possible, other fall-related risk factors still have not yet been explored, such as type and number of chronic diseases. Finally, because of the limitations of time, personnel, and material resources, this study surveyed only older adults in urban communities in Fuzhou, and the generalizability of the results may be limited. Future research can consider expanding the scope to different cities and carrying out multi-centre large-sample surveys to explore and compare fall prevention knowledge among older adults using random stratified sampling.

## Data Availability

The datasets generated and/or analyzed during the current study are not publicly available due this article is part of the author's master's thesis, her graduation thesis has a confidentiality period of 2 years, and other related papers have not yet been published. The datasets were not suitable for publication now but are available from the corresponding author upon reasonable request.
