# Correction: Development of the Home-Based Fall Prevention Knowledge (HFPK) questionnaire to assess home-based fall prevention knowledge levels among older adults in China

**DOI:** 10.1186/s12889-022-14761-x

**Published:** 2022-12-05

**Authors:** Yuting Yang, Qiong Ye, Miao Yao, Yongwei Yang, Ting Lin

**Affiliations:** 1 The School of Nursing, Fujian Medical University, Fuzhou, Fujian China; 2 Nursing Department, the First Afliated Hospital of Fujian Medical University, Fuzhou, Fujian China; 3 Ningde Municipal Hospital of Ningde Normal University, Ningde, Fujian China

**Correction: BMC Public Health 22, 2071 (2022)**

**https://doi.org/10.1186/s12889-022-14546-2**

The original publication of this article [1] contained an error in an equation in the “Evaluation index” section. The incorrect and correct equation are shown below.

**Incorrect**

- Cr=(C α + Cs)/3

**Correct**

- Cr=(C α + Cs)/2

This does not affect the conclusions/results of the article. The original article has been updated.
